# Social Justice in Dental Research

**DOI:** 10.1177/23800844231158258

**Published:** 2023-03-17

**Authors:** J.S. Feine

**Affiliations:** 1 Oral Health and Society Division, Faculty of Dentistry, McGill University, Quebec, Canada

**Keywords:** equity, inclusion, diversity, indigenous, space travel, periodontal disease

The renowned father of modern pathology, Rudolf Virchow, stated that “medicine is a social science, and politics is nothing else but medicine on a large scale” (Mackenbach 2009). Virchow’s beliefs stemmed from his experiences during the Silesian typhus epidemic in the mid-19th century after having observed the living conditions of communities most affected by the disease. Due to his belief that health is determined by one’s social conditions, his numerous essays and his subsequent political activities aimed to improve these determinants of health, he is also credited with being the father of social medicine (Lange 2021). The American Public Health Association (2023) describes these social determinants of health as follows: “The social circumstances in which we are born, live, and work play a greater role in longevity and overall health in the United States than genes, health insurance, and access to health services.” Because social determinants of health are primary factors associated with wellness and disease, it is politicians who are best positioned to facilitate social justice in health (Meili and Hewett 2016). Meili and Hewett (2016) emphasize that “if politicians truly did see themselves as the public’s physicians, we would have a far healthier society to show for it.”

Those of us who are not politicians can address social justice issues within our own domains. While we are likely to be cognizant of the concepts of equity, diversity, and inclusion, we may not know exactly how to incorporate these principles within our own research programs. In this journal issue, Fleming and Burgette (2023) provide us with 3 specific steps that we can take to “integrate and sustain” equity, diversity, and inclusion within our oral health research programs. In this very engaging commentary, they clarify the issues and describe practical steps that can readily be taken to enrich our research proposals, reduce bias, and communicate appropriately.

Of course, social justice must also extend to interactions between patients and their oral health care providers. Thus, educators have begun to include social justice principles within undergraduate dental education curricula. However, more research is needed to understand and address the barriers to equity in our dental students’ populations. To address this issue, Kontaxis and Esfandiari (2023) interviewed senior dental students, clinical instructors, and professors in a French-Canadian dental school on their perceptions of social justice and social justice education (SJE). Based on these interviews, the authors describe barriers to SJE, as well as administrative actions and teaching approaches that could lead to more effective social justice education outcomes (Kontaxis and Esfandiari 2023).

Beyond the realm of social justice (“in a galaxy far, far away”), this issue also includes an illuminating research report on space travel. Space travel will become even more possible for larger numbers of people in the not-so-distant future. Thus, understanding the impact of space travel on the craniofacial structures is essential. Based on a systematic review and meta-analysis, Moussa et al. (2023) analyzed changes in craniofacial hard tissues during flights in space. They found a trend toward increased bone volume/tissue volume (BV/TV) and tissue mineral density (TMD) in rodents, as well as a significant increase in TMD in humans in the roof of the skull. However, BV/TV significantly decreased in the mandibles of spacefaring rodents. Based on these findings, the authors suggest that space travel may engender unanticipated and varied changes in craniofacial bones; they also report that a significant research gap exists in our understanding of spaceflight-related alterations of craniofacial hard tissues (Moussa et al. 2023).

Back on earth, periodontal disease (also associated with hard tissue loss) is a topic of interest addressed within this issue. Merchant et al. (2023) used a large, US population-based sample of middle-aged and older adults to explore the association between IgG antibodies against periodontal microorganisms and cognition. They found that these antibodies were associated with lower cognition in people 60 y and older who had not previously been diagnosed with cognitive impairment. Thus, the authors suggest that periodontal disease may be used as a predictor of cognition. In another report on periodontal disease, Kaye et al. (2023) describe their analysis of the electronic health records of almost 3,500 US adults examined at a university dental clinic. In this retrospective cohort study, they found that overweight and obese patients were at greater risk for severe periodontal disease and that the treatments they received were more intense than for individuals at normal weights, independent of disease severity.

Also in this issue, Fernando et al. (2023) examined the sociodemographic status of Indigenous and non-Indigenous children in Australia and its association with toothbrushing frequency. Their findings suggest that, while sociodemographic status is associated with lower toothbrushing frequency in non-Indigenous children, this was not the case with Indigenous children. For Indigenous children, those with 1 parent born outside Australia were more likely to brush more frequently. These findings indicate that cultural differences that may also have an impact on oral health and disease exist within apparently homogeneous communities (Fernando et al. 2023).

Along the same lines, Suprabha et al. (2023) investigated the weaning routines used by parents of children with early childhood caries (ECC) living in India. Through parental qualitative interviews, they found that weaning practices carried out longer than necessary, as well as bedtime feeding, and intake of sugary drinks could explain the occurrence of ECC.

Other interesting articles can be found in this April issue, such as an investigation of oral health–related quality of life in Kenyan children with HIV (Wang et al. 2023), an assessment of the quality of current American Dental Association Clinical Practice Guidelines (London et al. 2023), and a survey of dental health care workers on their perceptions of vaccine safety and adoption of risk mitigation strategies (Coker et al. 2023). I hope you’ll enjoy the read!
